# Challenging the Boundaries of the Physical Self: Distal Cues Impact Body Ownership

**DOI:** 10.3389/fnhum.2021.704414

**Published:** 2021-10-14

**Authors:** Klaudia Grechuta, Javier De La Torre Costa, Belén Rubio Ballester, Paul Verschure

**Affiliations:** ^1^Synthetic, Perceptive, Emotive and Cognitive Systems Lab (SPECS), Institute for Bioengineering of Catalonia (IBEC), The Barcelona Institute of Science and Technology (BIST), Barcelona, Spain; ^2^Pompeu Fabra University, Barcelona, Spain; ^3^Institució Catalana de la Recerca i Estudis Avançats, Barcelona, Spain

**Keywords:** body ownership, active perception, embodied cognition, distal sensory cues, sensory prediction error, forward model

## Abstract

The unique ability to identify one’s own body and experience it as one’s own is fundamental in goal-oriented behavior and survival. However, the mechanisms underlying the so-called body ownership are yet not fully understood. Evidence based on Rubber Hand Illusion (RHI) paradigms has demonstrated that body ownership is a product of reception and integration of self and externally generated multisensory information, feedforward and feedback processing of sensorimotor signals, and prior knowledge about the body. Crucially, however, these designs commonly involve the processing of proximal modalities while the contribution of distal sensory signals to the experience of ownership remains elusive. Here we propose that, like any robust percept, body ownership depends on the integration and prediction across all sensory modalities, including distal sensory signals pertaining to the environment. To test our hypothesis, we created an embodied goal-oriented Virtual Air Hockey Task, in which participants were to hit a virtual puck into a goal. In two conditions, we manipulated the congruency of distal multisensory cues (auditory and visual) while preserving proximal and action-driven signals entirely predictable. Compared to a fully congruent condition, our results revealed a significant decrease on three dimensions of ownership evaluation when distal signals were incongruent, including the subjective report as well as physiological and kinematic responses to an unexpected threat. Together, these findings support the notion that the way we represent our body is contingent upon all the sensory stimuli, including distal and action-independent signals. The present data extend the current framework of body ownership and may also find applications in rehabilitation scenarios.

## Introduction

The sense of body ownership, which allows us to determine the boundaries between the own physical self and the external world, and therefore the source of a given sensation, is fundamental in adaptive goal-oriented behavior and survival (Engel et al., 2001; Tsakiris et al., 2006a; Tsakiris, 2010; Kilteni and Ehrsson, 2017). Indeed, during the last three decades, scientists have increasingly questioned both behavioral and neural mechanisms driving the emergence and experience of body ownership as well as its flexibility (Botvinick and Cohen, 1998; Ehrsson et al., 2004; Ernst and Bülthoff, 2004; Tsakiris, 2010; Blanke, 2012; Apps and Tsakiris, 2014). Together, the results support the notion that the way we perceive our body relies on an interplay between (1) bottom-up reception of self-generated (reafferent) and externally generated (exafferent) information from multiple sensory sources, (2) cerebellar-like feedforward and feedback processing of sensorimotor signals, and (3) comparison between the novel sensory stimuli and the priors about the body based on the history of sensorimotor contingencies (Botvinick and Cohen, 1998; Tsakiris and Haggard, 2005; Blanke, 2012; Apps and Tsakiris, 2014).

To date, at the empirical level, the principles underlying the representation of the body (in healthy population) have been studied using bodily illusions (Costantini, 2014). In these paradigms, the perception of one’s own body (i.e., the experienced body) is manipulated such that participants incorporate external objects or avatars to the representation of their body, which leads to a biased experience of the perceived body. A well-established experimental paradigm is the Rubber Hand Illusion (RHI), which is used to induce ownership over a rubber hand by manipulating the congruence of tactile cues externally delivered by an experimenter in the form of brush strokes (Botvinick and Cohen, 1998). Specifically, in this paradigm, the illusion of ownership is elicited while the subject observes the rubber hand being stroked synchronously (but not asynchronously) with the real hand occluded to vision. Curiously, RHI was found to co-occur with a disownership of the real hand. Another standard method for inducing ownership is the so-called moving Rubber Hand Illusion (mRHI). In mRHI, the visual feedback of voluntary arm or finger movements is either synchronized with the actual trajectory or not, thus biasing the experience of ownership over the virtual body (Tsakiris et al., 2006b; Dummer et al., 2009; Sanchez-Vives et al., 2010; Kalckert and Ehrsson, 2012). Crucially, both in the RHI and mRHI or their variations, one of the sensory signals manipulated to induce the experience of ownership always involves the processing of a proximal modality, which requires an object to enter in direct contact with the surface of the body, such as touch or proprioception (Botvinick and Cohen, 1998; Sanchez-Vives et al., 2010). Consequently, the current understanding of the mechanisms driving body ownership is constrained to the study of sensory cues that pertain to the body or the sensory consequences of voluntary self-initiated movements within the peripersonal space (PPS) (Rizzolatti et al., 1981; Holmes and Spence, 2004; Makin et al., 2007, 2008; Brozzoli et al., 2011a, b). Indeed, only recently it has been shown that, in the context of a goal-oriented action, such as hitting the puck in Air Hockey, body ownership is also modulated by cues processed by distal modalities sensing from a distance without getting in direct contact with the body (i.e., auditory, visual), provided that they pertain to the task and thus are task-relevant (Grechuta et al., 2019). Interestingly, however, none of the previous studies has addressed the question of whether distal task-irrelevant and action-independent signals which pertain exclusively to the environment contribute to the experience of body ownership. Hence, within the current body ownership framework their role remains elusive.

With seemingly no effort, we generate unambiguous interpretations about the self and the environment and determine the boundaries between them (Crapse and Sommer, 2008). To this aim, our brain uses multiple sources of sensory information processed by different modalities (i.e., vision, touch, audition) (Ballester et al., 2015; Grechuta et al., 2019). Based on this view, like any other coherent and robust percept, body ownership would require the merging of multisensory information which continuously occurs not only within but also outside of the body (Ernst and Bülthoff, 2004; Grechuta et al., 2019). Until now, however, the experimental evidence about the multisensory representation of the body and the necessary and sufficient conditions for the experience of its ownership is grounded exclusively in the study of proximal cues (Botvinick and Cohen, 1998; Tsakiris, 2010; Blanke, 2012; Ferri et al., 2017; Grechuta et al., 2017; Kilteni and Ehrsson, 2017). For instance, the seminal experiment of Botvinick and Cohen (1998), and later many others (for review see Tsakiris, 2010) who used RHI as a method to manipulate body ownership, proposed that the self-attribution of the rubber hand arises reactively as a result of bottom-up processes of combination and integration of information from visual and tactile modalities. Hence, initially, the illusion of owning the fake hand was interpreted as a passive perceptual state whose strength would be correlated with temporal discrepancies between seen and felt sensory stimuli (both necessary and sufficient). In the light of recent findings, however, this classical (Tsakiris, 2010) view on body ownership as resulting from perceptual correlations does not seem sufficient. For instance, it has been widely accepted that, despite its flexibility, in the context of externally generated sensory cues (e.g., tactile strokes as in the RHI), body ownership requires physical, anatomical, postural and spatial congruency of the real (felt) and fake (viewed) hands (Tsakiris and Haggard, 2005; Costantini and Haggard, 2007; Lloyd, 2007; Haans et al., 2008; Makin et al., 2008; Ferri and Costantini, 2016). These findings strongly suggest that the interpretation of the “novel” sensory evidence and possible incorporation of the rubber hand into the representation of the body is constrained by top-down prior knowledge updated by exposure and experience (Tsakiris and Haggard, 2005; Blanke, 2012; Apps and Tsakiris, 2014; Liepelt et al., 2017). In particular, the construct of ownership seems to rely on an internal model of the body that relates the physical aspects of the perceived rubber hand to the inputs received through a history of sensorimotor contingencies acquired through interactions of an agent with the world (Tsakiris, 2010; Apps and Tsakiris, 2014). Interestingly, this hypothesis is consistent with the Bayesian framework on perception (Engel et al., 2001; Kersten et al., 2004; Friston et al., 2010), which proposes that the bottom-up stream of sensory inputs coming from the senses is controlled by top-down cascade of neurally encoded hypotheses, the so-called priors, about the state of the body and world that are based on previous experience and generalized knowledge. As such, perception is a process whereby all acquired sensory information is continuously compared against experience-driven internal models of the self and the environment (Perrett et al., 1985; MacKay and Crammond, 1987; Carlsson et al., 2000; Brown and Brüne, 2012; Ferri et al., 2017). A mismatch between the predicted and the actual inputs results in a prediction error, which triggers the system to revise the likelihood of its hypotheses. This framework can explain why, during RHI, it is possible to embody the rubber hand but not other objects that do not have anthropomorphic shapes, despite visuotactile correlations (Seth, 2013; Apps and Tsakiris, 2014).

Grounded in the framework of active perception, Ferri et al. (2013, 2017) studied whether body ownership can be modulated by an expectation of exafferent tactile feedback in the absence of actual physical touch of either the fake or the real body-parts. Interestingly, their experiment revealed that a mere expectation of an upcoming sensory event, predicted by an anticipatory response in multisensory parietal cortices, is indeed sufficient to induce the experience of ownership over a rubber hand, measured subjectively and objectively (Ferri et al., 2013, 2017) (see also Smit et al., 2018). This finding supports the notion that body ownership can emerge as a result of the pure expectation of correlated multisensory inputs, which challenges the originally defined necessary conditions of the embodiment discussed above (Tsakiris, 2010). In the present study, we extend this hypothesis and propose that, like any coherent percept, body ownership is a result of bottom-up integration and top-down prediction of all the sensory stimuli processed by, interoceptive and exteroceptive, proximal and distal modalities including those which pertain to the environment. Hence, we propose that body ownership may also depend on the consistency of distal sensory signals which occur in the environment, even when they are independent of volitional actions. Additionally, we believe that enhanced ownership experience may positively impact motor behavior.

To test our hypothesis, we created an embodied goal-oriented task using virtual reality and manipulated the predictability of the surrounding events in the environment by changing its rules while preserving proximal and action-driven signals congruent. We predicted that body ownership of a virtual avatar will be negatively influenced in the condition where external sensory signals underlying the statistical structure of the environment are not predictable.

## Materials and Methods

### Participants

We recruited twenty-four students from Universitat Politècnica de Catalunya. To determine the sample size, we computed a power analysis based on self-reported ownership data from a previous experiment. The analysis yielded that a minimum of 16 participants was required for an α of 0.05 and the power of 0.8. Furthermore, we computed a non-parametric sensitivity analysis (Wilcox, 2019) to assess the effect size in each of the main reported outcomes. This analysis yielded medium size effect (Wilcox *Q* = 0.422) for the self-reported ownership measures and medium-to-large effect (Wilcox *Q* = 0.68) for the physiological response (i.e., Galvanic Skin Response, GSR), which lead us to rely on the between-groups differences found in the experimental setup. For this study, following an eligibility interview, we included healthy, right-handed (handedness assessed using the Edinburgh Handedness Inventory; Oldfield, 1971) participants with normal hearing, normal or corrected-to-normal vision, and no previous experience in a body-ownership study: 12 males (Mean ± SD 23.25 ± 2.37 years-old) and 12 females (Mean ± SD 22.16 ± 2.12 years-old). We performed stratified randomization in order to ensure the balance of the experimental conditions with respect to age, gender and previous experience with virtual reality. Similar to Armel and Ramachandran (2003), a between-subjects experimental design was chosen to prevent the maturation bias (Slack and Draugalis, 2001) and, specifically, habituation to the ownership measures and sensory manipulations. The experimental procedures were previously approved by the ethical committee of the University of Pompeu Fabra (CIREP-UPF, Barcelona, Spain).

### Experimental Setup

**FIGURE 1:**
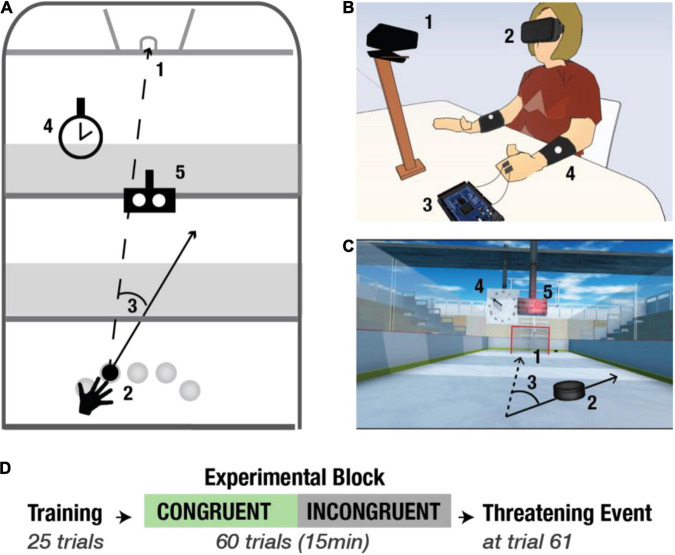
Experimental setup and protocol. **(A)** Schematic bird view of the virtual environment. 1- goal; 2- five starting positions; 3- an example of a directional error; 4- a clock; 5- the Go Signal (GS). **(B)** Experimental setup. 1- motion tracking device (Kinect); 2- Head Mounted Display (HMD, HTC-Vive Headset); 3- Arduino and E-Health; 4- Bracelets with reflecting markers for the tracking. **(C)** Virtual scene. The first-person perspective view of the scene. **(D)** Experimental protocol. All participants go through the Training Block (TB) (25 trials). Then they are randomly split into two experimental conditions: congruent (congruent condition, green arrow) or incongruent (incongruent condition, black arrow), and both groups undergo the Experimental Block (EB). At the end of the experiment, all the participants experience the Threatening Event.

Similar to previous experiments (Makin et al., 2008; Grechuta et al., 2017), here, we used the virtual reality method as a tool to investigate the modulation of body ownership (MacKay and Crammond, 1987). Such as in Grechuta et al. (2019), the experimental protocol was implemented using the Rehabilitation Gaming System (RGS) interface, comprised by a personal computer, a head-mounted display (HTC Vive),^1^ a motion detection input device (Kinect, Microsoft, Seattle, United States), and active noise control headphones (Beats Electronics LLC, California, United States) (Figures 1A,B). The Virtual Environment (VE) was designed using SketchUp (Trimble Inc., California, United States) (Figure 1C), and the software was developed in Unity3D (Unity Technologies SF, Copenhagen, Denmark, version 2018.2.5f1). During the experiment, participants sat at a table with their arms resting (Figure 1B). All subjects were seated in the same static chair. The position of the chair was the same for all participants. However, they were allowed to rotate their body and move their head freely to explore the virtual environment. The movements of the arms (extracted from the position of the shoulders, elbows and forearms indicated by reflecting markers, Figure 1B4), were continuously tracked and mapped onto the avatar’s arms enabling interaction with the VE. Fingers were neither tracked nor animated.

### Virtual Air Hockey Task

The objective of each session was to complete a goal-oriented motor task that consisted of hitting a virtual puck into a goal (Figure 1A). Prior to the experiment, participants received instructions to place their hands in square-shaped Go Areas (GA; one for the left and one for the right hand) at the beginning of every trial. The trial started, and the puck appeared only when the system detected that both hands were in the GAs. Importantly, although both hands were mapped and rendered in the virtual scene, the task was to be completed using the right arm exclusively. To counteract repetitive movement-patterns, prevent habituation, and ensure that participants pay attention to the task, the puck was spawned pseudorandomly in one of the five Starting Positions (SP), such that the same SP did not repeat on consecutive trials. SPs were distributed evenly within the right-hand workspace (Figure 1A2). The game was designed such that the puck did not bounce against the walls. Thus, a trial consisted of one-hit only which could end in either a success (i.e., the puck enters the goal) or a failure (i.e., the puck hits one of the walls). Both events were immediately indicated by auditory feedback in the form of semantically corresponding sound, positive or negative, respectively. The puck was visible throughout the experiment. Images of the arm and hand, respectively, are presented in the Supplementary Material.

### Protocol and Sensory Manipulations

The experiment consisted of three parts (Figure 1D), including (1) Training Block (TB) (25 trials), (2) Experimental Block (EB) (60 trials), and (3) Threatening Event (1 trial). TB, in particular, allowed familiarization with the virtual environment as well as with the dynamics of the game. Threatening Event served to record autonomous, physiological responses to an unexpected threat, which we used as an objective measure of body ownership (Armel and Ramachandran, 2003). To investigate whether sensory cues that pertain to the environment influence body ownership, in the EB, participants were randomly split into two groups: congruent condition (green), and incongruent condition (black) (Figure 1D). We exposed the incongruent group to a lower congruency (i.e., lower predictability) of visual and auditory action-independent and task-irrelevant cues. The Congruent group served as control.

The virtual environment consisted of (1) the virtual arms viewed from the first-person perspective, (2) an Air Hockey field, (3) the puck, (4) a goal, (5) benches for the audience, (6) the Go Signal (GS), and (7) a clock (Figures 1A,C). The virtual solar time was indicated by the position of the sun in the sky (i.e., visual cue), while the location (i.e., the setting) was signaled by the background sound representative for a given place (e.g., background sound of the air-hockey field, concert, or cinema) (Figure 1C). The default scene was set at midday on a hockey field, the benches surrounding the pitch were still, and the clock indicated the actual time. Both in the TB (identical for both conditions) and in the EB of the congruent condition the behavior of all the scene components mentioned above, as well as the time of the day and location, was always the same and, therefore, congruent and entirely predictable. Moreover, in both conditions, all the auditory and visual signals relevant to the body (within the PPS) and to the task (i.e., Air Hockey field, the puck, the goal, and the Go Signal, the trajectory of the puck, the outcome of the action) were always congruent. Crucially, in the EB of the incongruent condition, we introduced manipulations of task-irrelevant signals, such as the time of the day and the location as well as the default behavior of different scene components including benches and the clock. First, the virtual time was changed by either altering the position of the sun in the sky or by replacing it with the moon. Second, the virtual location shifted by changing background music. For example, the sound would change from a sound representative for an Air Hockey pitch to an audio representative for a concert. Third, the orientation of the benches, which surrounded the pitch, rotated on the *z*-axis. Finally, the behavior of the clock altered. This manipulation was achieved by slowing down or speeding up the velocity of the arrows indicating the time or by changing their direction (i.e., from clockwise to anticlockwise). Importantly, to ensure that the sensory manipulations in the incongruent condition impact exclusively the perception of the environment and not the action, they were always introduced randomly between the end of a trial and the beginning of the consecutive one. Moreover, each of the manipulations was triggered only once per trial at a random time within a 2 s time-window after the trial ended (i.e., when the puck either entered the goal or hit one of the walls) and the next trial started. The incongruencies were introduced gradually, and they were pseudo-randomly distributed (i.e., there was one manipulation present at a time, and it did not repeat consecutively). This design prevented the attribution of action-driven causality to the emergence of the experimental manipulations. Critically, in both conditions, all the task-relevant stimuli including visual feedback of the arms and auditory performance feedback (i.e., positive and negative sound) were always congruent with the initiated action and, therefore, entirely predictable. Additionally, to counteract possible attentional biases caused by the experimental manipulations in the incongruent condition, before the experiment all participants received the following instructions: the main objective of this experimental session is to attend to the motor task and to do your best to score the maximum number of goals.

### Measures

#### Embodiment Measures

We used (1) body ownership questionnaire, (2) physiological responses to an unexpected threat, and (3) hand withdrawal kinematics as a subjective, objective, and behavioral proxy to the embodiment of the virtual avatar. To assess the conscious experience of body ownership, we administered a 6-item questionnaire adapted from previous studies (Longo et al., 2008; Kalckert and Ehrsson, 2012), whereby three questions are related to the strength of the illusion, and the remaining serve as controls. Answers for each statement were provided on a 7-point Likert Scale ranging from “−3”: being in strong disagreement to “3”: being in strong agreement. To counteract order effects, the sequence of questions was randomized between participants. The questionnaire was administered after the Threatening Event at the end of the experiment.

Galvanic Skin Responses were recorded as the physiological measure of the autonomous nervous system, which increases as a reaction to arousing stimuli. Similar to other studies (Armel and Ramachandran, 2003), in the present protocol, we used GSR as an objective quantification of the ownership illusion. In particular, at the end of the EB, in each condition, we computed GSR responses to an unexpected threat (i.e., a knife falling to stab the right virtual hand). Post-Threatening Event signals per each participant were normalized by subtracting the mean signal from all the experiment before the stimulus onset. To prevent movement-driven muscular artifacts, we recorded GSR from the left hand, which was at rest during the experiment. The signal was recorded using an Arduino e-Health board at a sampling rate of 33 Hz from two flat reversible silver/silver chloride (Ag–AgCl) electrodes which were attached to the middle and index fingers, respectively (Figure 1B3). To compute the event-specific responses exclusively while discarding possible confounding factors, such as fatigue, the data were preprocessed to extract phasic components from tonic activity based on Continuous Decomposition Analysis (CDA) (Benedek and Kaernbach, 2010) as implemented in the Ledalab software (Leipzig, Germany). For the analysis, we computed the number of significant (i.e., above 0.01 mS threshold) phasic skin conductance responses (nSCR) within the entire response window that lasts from the stimulus onset to the end of the experiment (i.e., between 1 and 12 s) and compared the number of those responses between the two conditions. Finally, as a behavioral measure of embodiment, we computed the velocity of hand withdrawal following the Threatening Event. To this end, we collected kinematic movement data from the Kinect for each participant throughout the experiment. All the data from the system were recorded at 33 Hz. To quantify the execution of instinctive defensive movements, such as hand withdrawal in response to the unexpected Threatening Event (i.e., the virtual knife stabbing the virtual hand) (González-Franco et al., 2014), we computed the cumulative velocity of displacement of the right virtual hand. The results were compared between the two conditions.

Due to possibly stronger assimilation of the virtual hand to the representation of the body, we expected significant differences on all ownership measures between conditions. Specifically, we predicted that the self-reported scores, GSR responses, and kinematics would be higher in the congruent as compared to the incongruent condition suggesting stronger ownership.

#### Control Measures

In virtual environments, the sense of presence refers to the subjective experience of “being there,” despite the physical distance. In particular, when a user does not perceive the influence of technology during a virtual reality-based experience (Witmer and Singer, 1998; Sanchez-Vives and Slater, 2005). To ensure that the participants in both groups felt equally immersed within the proposed environment, after the experiment, we asked them to complete a presence questionnaire by assessing each of the items on a 7-point Likert Scale. Following that, to ensure that all participants experienced control over the virtual avatar, we administered an agency questionnaire (Longo et al., 2008; Kalckert and Ehrsson, 2012), which consisted of six items in total, three of which served as controls. Participants were required to answer each statement on a 7-point Likert Scale. All items from the presence and agency questionnaires are shown in the Supplementary Material. We predicted that the sensory manipulations introduced in the incongruent condition would not impact either presence or agency scores. Hence, we expected the experience of presence agency in the incongruent condition.

#### Performance

To evaluate performance in both conditions, we measured scores, angular errors, as well as reaction and response times, all stored by the system. Scores were calculated as the percentage of successful trials, namely, the times when the puck entered the gate (Figure 1A1). An angular error was computed as the difference between the actual direction vector and a straight line between the starting position of the puck and the middle of the goal (desired trajectory, Figure 1A3). Reaction times were the time intervals between the apparition of the puck and the moment of “leaving” the starting position to hit it, while the response times were the time intervals between the apparition of the puck and the moment of its collision with the hand. The evaluation of performance allowed us to understand whether, in both groups, participants paid attention to the task. Based on previous studies (Grechuta et al., 2017) we predicted that the experimental manipulations in the incongruent condition may performance such that it is lower than in the congruent condition.

## Data Analysis

The statistical analysis followed non-parametric methods. This choice was determined by the skewness of distributions as computed with the D’Agostino-Pearson test. Those measures included the ownership questionnaire (*p* < 0.001), SCRs (*p* < 0.05), and hand withdrawal (*p* = 0.01). Hence, we used the Mann-Whitney *U* test for between-groups analyses and the Wilcoxon signed-rank test for within groups comparisons. All comparative analyses used two-tailed tests and a standard level of significance (*p* < 0.05). The data were analyzed using Python3.7^2^ and Matlab (Mathworks, Natick, United States, version 2018a).

## Results

### Presence and Agency

First, we performed analyses of the three control measures. The results of the perceived experience of presence revealed that in both conditions participants indeed felt immersed in the proposed virtual environment {congruent condition: μ = 1.6 (*CI* = [1.39, 1.78]), *SD* = 1.56 and incongruent condition: μ = 1.51 (*CI* = [1.29, 1.73]), *SD* = 1.8}. No differences were found between the groups (*U* = 34757, *p* = 0.47). Second, we found that participants in both the congruent {μ = 1.19 (*CI* = [0.76, 1.62]), *SD* = 1.24} and the incongruent group {μ = 1.3 (*CI* = [1.1, 2.0]), *SD* = 1.3} reported strong perceived agency such that in both groups the mean score was higher than 1.1 (Haggard, 2017). No differences were found between the groups for either the experimental (*U* = 531, *p* = 0.08) or the control agency questions (*U* = 518, *p* = 0.1).

### Embodiment

**FIGURE 2:**
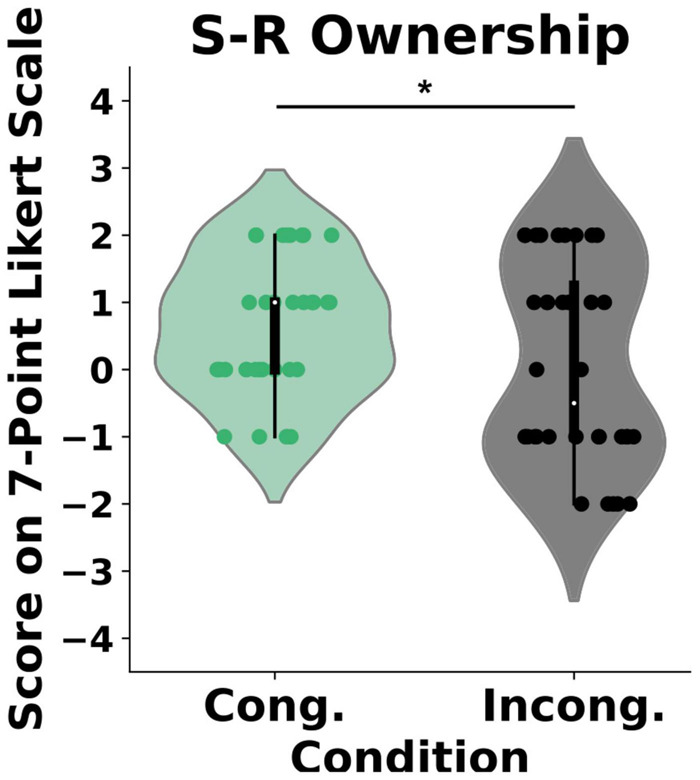
Self-reported experience of ownership. *Y*-axis: Responses on the 7-point Likert scale ranging from “−3” (strongly disagree) to “3” (strongly agree). Scores above “0” indicate a feeling of body ownership. *P < 0.0.

**FIGURE 3:**
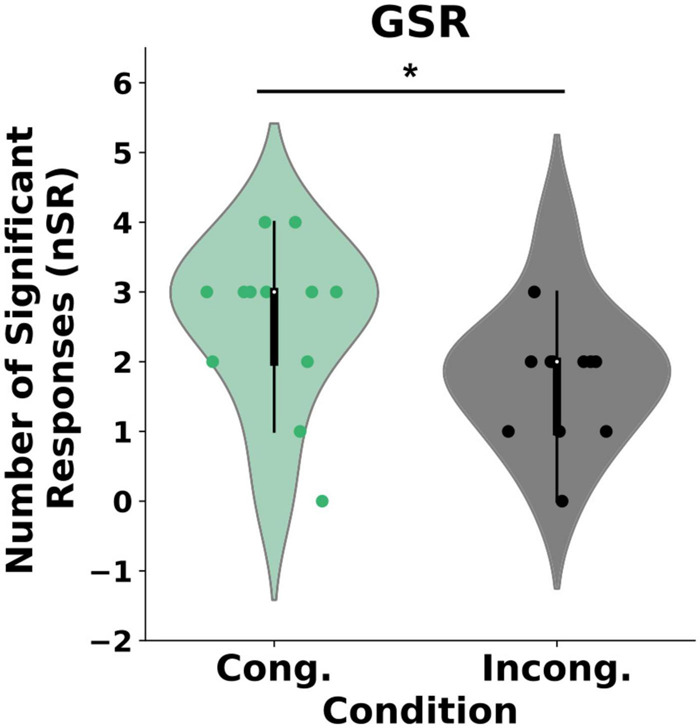
GSR results. The plot represents the difference between the groups in the number of galvanic skin responses (nGSR) post stabbing event. *P < 0.0.

Our analysis revealed a statistical difference in the self-reported experience of body ownership between the two conditions (*U* = 500, *p* = 0.04) such that the participants perceived significantly stronger ownership in the congruent condition {μ = 0.6 (*CI* = [0.27, 0.94]), *SD* = 0.9} than in the incongruent condition {μ = 0.05 (*CI* = [−0.44, 0.55]), *SD* = 1.45}, Wilcox *Q* = 0.422 (medium effect size, Figure 2). Importantly, we found no differences in the control questions between the groups (*U* = 535, *p* = 0.1). To further explore the effects of the congruency of external cues on body ownership, we computed post-threatening GSR responses for every individual in both the congruent and the incongruent condition (Figure 3). As expected, and in line with the literature, the GSR signal increased in both groups. Specifically, the Threatening Event triggered a significant increase in the number of galvanic skin responses in the congruent condition {pre-TE: μ = 1 (*CI* = [0.61, 1.38]), *SD* = 0.577, post-TE: μ = 2.58 (*CI* = [1.84, 3.32]), *SD* = 1.11, *p* = 0.0008} and in the incongruent condition {pre-TE: μ = 1.16 (*CI* = [0.79, 1.53]), *SD* = 0.55, post-TE: μ = 1.83(*CI* = [1.17, 2.48]), *SD* = 1.64, *p* = 0.03}. Crucially, however, we found a statistically significant difference between the groups in the numbers of activations post-Threatening Event (*U* = 41, *p* = 0.003) (see section “Materials and Methods”) such that the number was significantly higher in the congruent condition {μ = 2.58 (*CI* = [1.84, 3.32]), *SD* = 1.11} than in the incongruent condition {μ = 1.833 (*CI* = [1.17, 2.48]), *SD* = 0.98}, Wilcox *Q* = 0.68 (medium-to-large effect size, Figure 3).

**FIGURE 4:**
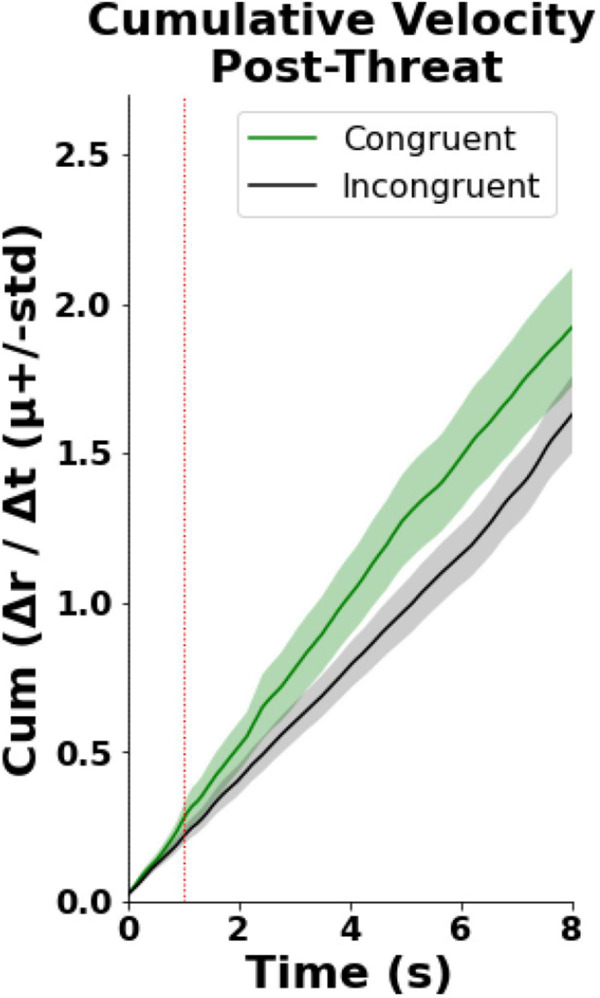
Hand withdrawal results. T0 indicates the time when the knife appeared while the dashed red line shows the time when the knife stabbed the hand (time = 1).

Finally, we observed that in the congruent condition participants exhibited faster cumulative velocity of the right virtual hand displacement post Threatening Event (hand withdrawal, Figure 4). In particular, the statistical analysis revealed a statistically significant difference between the incongruent {μ = 1.00 (*CI* = [0.78, 1.22]), *SD* = 0.33} and congruent {μ = 1.31 (*CI* = [0.95, 1.68]), *SD* = 0.54} groups [*U* = 43, *p* = 0.04, Wilcox *Q* = 0.65 (medium-to-large effect)] in the averaged cumulative sum of the virtual hand velocity (Figure 4). This analysis was modified due to a reviewer’s request during the review process. Please, see the Supplementary Material for the original analysis.

To evaluate the reliability of the three measures of ownership included in the study, we have analyzed their internal consistency using Cronbach’s alpha. The analysis yielded a value of 0.68 supporting the consistency of the implicit and explicit dimensions of ownership evaluation. Together, the significant differences (Mann-Whitney *U*) found in the evaluated ownership dimensions (explicit, implicit) and the effect sizes found for these metrics suggest that the incongruencies in distal sensory modalities influenced the experience of body ownership for the virtual avatar in the experimental as compared to the control condition.

### Performance Measures

**FIGURE 5:**
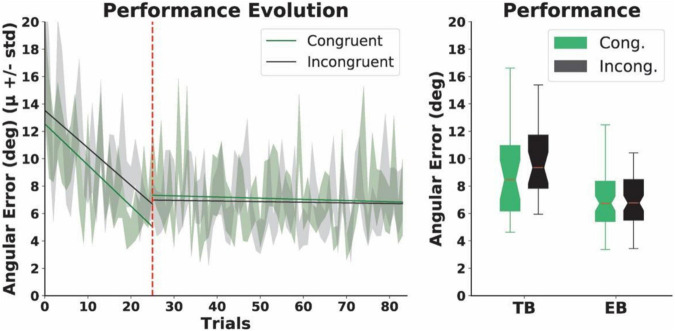
Motor performance. Left: the evolution of angular errors. The dashed red line indicates the end of the Training Block (TB) and the beginning of the Experimental Block (EB). The solid lines represent linear regression models for the angular errors in each condition in the TB and EBs, before or after the dashed red line, respectively. Right: total angular errors. Boxplots represent angular errors for the two conditions in the training and EBs, respectively. No differences were found between the groups.

Our results revealed that the normalized performance-scores (i.e., the proportion of successful trials) in both the congruent (μ = 0.6, *SD* = 0.17) and the incongruent group (μ = 0.59, *SD* = 0.18) ranged between 60 and 80% during the EB. The scores did not differ between the groups (*p* = 0.5). Subsequent analyses of the angular errors (Figure 5) showed that, during the TB, accuracy significantly improved in both groups. Specifically, we found that the angular errors significantly decreased from early to late trials both in the congruent (early trials: μ = 12.63, *SD* = 5.25 and late trials: μ = 6.11, *SD* = 3.76; *p* = 0.002) and the incongruent condition (early trials: μ = 14.03, *SD* = 6.9 and late trials: μ = 7.53, *SD* = 2.48; *p* = 0.004). We found, however, no within-group differences for the early and late trials in the EB, whereby participants in the experimental condition experienced sensory manipulations. Specifically, we report no differences for either the congruent (early trials: μ = 8.05, *SD* = 3.81 and late trials: μ = 7.14, *SD* = 4.87; *p* = 0.53) or the incongruent condition (early trials: μ = 7.1, *SD* = 3.85 and late trials: μ = 7.26, *SD* = 3.94; *p* = 0.48). Additionally, the Mann-Whitney *U* analyses yielded no differences in the mean angular errors between the groups in either the TB (*p* = 0.15) or the EB (*N* = 12, *p* = 0.15) (Figure 5). Finally, we report no differences in either response (congruent condition: μ = 2.35, *SD* = 0.78 and incongruent condition: μ = 2.43, *SD* = 0.67; *p* = 0.1) or reaction times (congruent condition: μ = 1.01, *SD* = 1.41 and incongruent condition: μ = 1.63, *SD* = 1.14; *p* = 0.3) between the two conditions during the EB. Although our performance estimates were sensitive enough to capture learning-derived changes during the TB only, we were not able to observe differences in performance between the groups during the Training and neither during the EB, therefore suggesting that the proposed manipulation of action-independent sensory signals in the incongruent condition may not have an effect on performance.

## Discussion

The unique ability to recognize one’s own body, experience it as our own, and localize it in space lies in the continuous processing of self- (i.e., reafferent) and externally generated (i.e., exafferent) multisensory information arising from sensorimotor interactions of an agent within the environment (Clark, 1999; Kawato, 1999; Wolpert and Flanagan, 2001; Crapse and Sommer, 2008). Vast evidence has now demonstrated that this processing comprises reception or sensory stimuli, feedforward and feedback processing of sensorimotor signals which pertain to the body or the task at hand and occur within the PPS, and prior knowledge about the body (Botvinick and Cohen, 1998; Maravita and Iriki, 2004; Sanchez-Vives et al., 2010; Ferri et al., 2013; Ballester et al., 2015; Serino et al., 2015; D’Angelo et al., 2018; Grechuta et al., 2019). The present study extends prior findings by showing that the plasticity of body ownership also depends on the consistency of body- and action-independent sensory cues which pertain to the environment and are processed by distal modalities (i.e., visual and auditory). Thus, for the first time, we empirically reveal that both the phenomenological percept (i.e., measured objectively using physiological responses and hand withdrawal) and metacognitive construct of body ownership (i.e., measured subjectively using self-reports) are contingent upon all the sensory stimuli including task-irrelevant signals occurring outside the PPS. We interpret our results from the perspective of the Bayesian framework of perception and propose that, similar to any robust percept, body ownership depends on the consistency of the internal models of not only the body or the consequences of its actions but also the consistency of the model of the surrounding environment (Wolpert and Miall, 1996; Engel et al., 2001; Ernst and Bülthoff, 2004; Crapse and Sommer, 2008).

A large body of evidence demonstrates that the experience of ownership emerges actively through dynamic comparisons between integrated and predicted multisensory signals (Seth, 2013; Suzuki et al., 2013; Apps and Tsakiris, 2014; Ferri et al., 2017; Grechuta et al., 2019). The influence of top-down processing (Suzuki et al., 2013) on the sense of ownership is supported in the contexts of classical RHI where the stimuli are externally generated (Botvinick and Cohen, 1998). In the RHI paradigm, self-attribution of the rubber hand arises actively as a consequence of the minimization of prediction errors resulting from multisensory conflicts during the synchronous stroking of the real and fake body-parts (i.e., visuotactile) (Apps and Tsakiris, 2014). Similarly, a failure to experience ownership over a non-corporeal object (Tsakiris et al., 2010) or a rubber hand located in an implausible position (Costantini and Haggard, 2007) can also be interpreted as a consequence of predictive matching of the sensory inflow and the experience-driven internal models of the body (i.e., priors) (Apps and Tsakiris, 2014). For example, the less plausible is the shape or the position of the rubber hand, the less likely is the hypothesis that the rubber hand belongs to me (Apps and Tsakiris, 2014).

The contribution of feedforward and feedback processing of sensorimotor signals in the emergence of body ownership is supported in the context of self-generated cues, such as in the mRHI paradigm (Sanchez-Vives and Slater, 2005; Dummer et al., 2009). Specifically, it has been proposed that the location of different body-parts is estimated by the central nervous system *via* a forward model or a corollary discharge, which generates predictions about the sensory consequences of movements and compares them with the corresponding sensory feedback (Wolpert et al., 1995; Miall and Wolpert, 1996; Sommer and Wurtz, 2008). Those predictions are carried out by the so-called efference copy that employs all the sensory signals relevant to the body and the goal of the task (i.e., task-relevant) (Sperry, 1950; Griisser, 1995; Wolpert et al., 2011). Sensory prediction errors from multiple sensory sources, which reflect the discrepancies between the expected and the actual sensory stimuli, inform about the state of the environment and the body, shaping the experience of ownership. For instance, when the visual feedback of the virtual hand does not match the expected one (e.g., the hand follows a different trajectory than the executed one) the prediction errors pertaining to the body increase resulting in a decreased experience of ownership over the virtual avatar (Dummer et al., 2009; Sanchez-Vives et al., 2010). Interestingly, even distal auditory consequences of self-generated movements can bias the experience of ownership provided that they are relevant to the task, thus informing about the magnitude of the error (Grechuta et al., 2019).

Hence, the evidence discussed above suggests that body ownership is compromised when the actual sensory signals violate the expected cues independently of whether they are externally (RHI) or self-generated (mRHI). The following question may then arise: if body ownership depends on the matching between the predicted and the actual sensory stimuli, can it, in a similar way, be affected by prediction errors about the sensory signals which are task-irrelevant, and which pertain to the environment? To answer this question, we designed a virtual reality-based paradigm where participants were to complete a motor task (Air Hockey) and manipulated the predictability of the external cues by randomly changing the rules of the environment. Thus, similar to the prediction errors which result from visuotactile matching and affect the generative model of the body (Botvinick and Cohen, 1998), or those which result from visuomotor (Sanchez-Vives et al., 2010) or visuoauditory (Grechuta et al., 2019) matching and affect the forward model, here we experimentally induced prediction errors which result from visuoauditory matching and affect the generative model of the environment (Friston et al., 2006; Clark, 2013). We expected that if body ownership depends on the congruency across all sensory modalities, it will be impacted in the EB of the incongruent condition where the expectations about the model of the environment acquired during the TB are violated. Our findings establish that incongruencies in action-independent and task-irrelevant sensory cues, which inform about the statistical structure of the environment and are processed by distal modalities, modulate the experience of body ownership. In particular, we found that the congruent as compared to the incongruent environment led to an enhanced experience of ownership over the virtual hand, as measured subjectively by a questionnaire (Figure 2), objectively through the galvanic skin responses (Figure 3), and behaviorally using the hand withdrawal (Figure 4). Crucially, we found that both groups obtained high scores on the control measurements. First, we found that, despite the introduced manipulations, participants in the incongruent condition perceived the environment as overall immersive and plausible (Sanchez-Vives and Slater, 2005). Second, the analysis of the agency questionnaire revealed that subject experienced agency over the virtual avatar in both conditions (mean score > 1.1; Longo et al., 2008). Strong agency in the incongruent group possibly occurred due to the lack of visuomotor (Haggard, 2017) or other task-relevant signals-manipulations (Grechuta et al., 2019). Finally, in both the congruent and the incongruent conditions, participants maintained 60–80% of success rate throughout the EB. Together these results support the notion that the experimental manipulations did not impact the perceived presence of the virtual scenario, agency or attention. We note, however, that, to explicitly and objectively control for possible attentional biases, future studies that manipulate perceptual stimuli, such as the present one, should include eye-tracking technology, which allows for a highly detailed analysis of gaze in ongoing tasks.

Grounded in the implicit and explicit measures of ownership, we propose that the violation of expectations in the context of the proposed paradigm can be understood as a sudden increase of uncertainty in the internal model of the environment. Consequently, a significant enough violation of expectations influenced by the sensory manipulations in the incongruent condition would have a global effect on modulating uncertainty in a range of cognitive functions (Craig, 2002; Tsakiris et al., 2007; Gentile et al., 2013). From a functional perspective, such sensory prediction errors would have an impact on all predictive models inducing uncertainty in both the model of the environment (i.e., a generative model) and the model of the body. In other words, the temporal modulation of global uncertainty would inevitably change the overall confidence in the internal model of the body as supported by our results (Figures 2–4). Possibly, however, depending on the source of the uncertainty (i.e., prediction error in proximal vs. distal cues) the impact on different models might be weighted differently such that e.g., (1) reafferent cues impact ownership stronger than exafferent ones [i.e., the ownership is generally stronger in the mRHI/vRHI that the classical RHI (Kokkinara and Slater, 2014; Kokkinara et al., 2016)], (2) task-relevant errors resulting from self-generated proximal sensory information impact self-model (i.e., body ownership) stronger than distal one (Grechuta et al., 2019), and (3) task-relevant errors impact ownership stronger than task-irrelevant cues (present results). This would explain why present self-reported ownership scores are relatively lower than those reported in the classical RHI or mRHI/vRHI. The proposed interpretation of the present findings is also consistent with the accounts of Bayesian causal inference according to which if my model of the environment is likely, the model of myself is likely too, and vice-versa (Körding and Wolpert, 2004; Doya et al., 2007; Apps and Tsakiris, 2014; Samad et al., 2015).

An interesting question which one could raise, however, is how persistent this effect is? Is it transient? It has been demonstrated that one way to minimize prediction errors is to update the current model, in our case, the model of the environment, to accommodate the unexpected sensory signals (Körding and Wolpert, 2004; Friston et al., 2010). This would suggest that prolonged exposure to random errors would lead to a subsequent reduction of uncertainty in the model of the environment reducing the neuromodulatory response, which, in turn, would reduce the uncertainty in the model of the body. The consequent increase in the reliability of the predictive models of the environment and the body would immediately result in the reestablishment of body ownership. In particular, we expect that after more prolonged exposure to the incongruent stimuli in the incongruent condition, the experience of ownership, measured both objectively and subjectively, would most probably return to normal such that there would be no differences in the perceived ownership between the two conditions. Further work shall systematically address this question by running additional trials to assess the temporal evolution of body ownership in the context of an incongruent environment. Similar, to improve the quality of the present pilot experiment and further support current findings, future studies will consider a bigger sample size and alternative objective measures of the sense of ownership (i.e., body temperature), which would allow a within-group design preventing from habituation to physiological signals measures (e.g., the threatening event).

What about the experience of control and performance? Our results demonstrate that, despite the presence of sensory manipulations in the incongruent condition in the EB, participants reported strong experience of agency and, contrary to what we expected, maintained 60–80% performance accuracy, as measured through scores, angular errors (Figure 5). On the one hand, we designed the paradigm such that all the proximal (i.e., within the PPS), action-driven (reafferent) and task-relevant stimuli were always congruent and therefore entirely predictable. Specifically, the 1:1 mapping between the real and the virtual hands ensured that the visual feedback of the movements in virtual reality reliably reflected the movements of the real hands resulting in visuomotor congruency which underlies agency (D’Angelo et al., 2018). In our experiment, similar to the peripersonal signals, the consequences of actions in the extrapersonal space signaled by auditory and visual feedback reflected real-world physics and were fully predictable in both conditions. That is, the sound of the puck temporarily and spatially corresponded to the location of its collision with the environment (i.e., the walls or the goal). On the other hand, we also experimentally controlled for the occurrence of the sensory manipulations to ensure that they were action independent. Specifically, they were always introduced randomly between the end of a trial and the beginning of the next one. Finally, neither did the manipulated signals in the incongruent condition inform about the outcomes of the task (i.e., knowledge of performance) nor did they affect motor performance, which makes them task irrelevant. Indeed, the goal of every experimental session was to complete the motor task as accurately as possible by hitting the puck into the goal. We thus propose that the prolonged exposure to the congruency (and therefore the predictability) of task-relevant visuomotor (i.e., proximal) stimuli and high performance-scores that are the key contributors to the experience of agency (Haggard, 2017) in both conditions reduced the uncertainty in the models of the proximal sensorimotor contingencies thus positively reinforcing the experience of agency, that is the experience of controlling one’s actions, and, through them, events in the outside world (Wolpert et al., 2011; Haggard, 2017). This interpretation is in line with the comparator model of agency whereby a sense of agency is generated when voluntary actions match outcomes (Haggard, 2017). Following the Bayesian interpretation of body ownership, we hypothesize, however, that deficits in the ownership experience might have impacted agency at the beginning of the EBs when the exposure to sensory matching was still low. Current design does not allow us to answer this question. Using repeated measures of agency evaluation would allow for shedding light on this hypothesis.

It is noteworthy that some recent studies observed a functional link between body ownership and performance. For example, in a previous study, we reported a significant correlation between the degree of ownership of a virtual limb, induced through a virtual RHI, and reaction times on a simple sensorimotor task (Grechuta et al., 2017). Grounded in this finding, we initially expected that performance in the incongruent condition would be impaired as a consequence of reduced ownership, which is not reflected in our results. Our interpretation is twofold. On the one hand, the two studies differ significantly with respect to the number of trials and, therefore, the overall time of the illusion induction such that in (Grechuta et al., 2017) the RHI lasted 25 min (150 trials) and, here, subjects experienced the manipulation for 10 min (60 trials). As such, the time might not have been enough for the increased ownership in the incongruent condition to influence performance. On the other hand, the two experiments employ diverse methodologies. In particular, in Grechuta et al. (2017), ownership is manipulated through synchronous or asynchronous stroking, whereby the illusion is induced or not, respectively. In the present study, participants experience ownership of the virtual avatar as a consequence of visuomotor correlations by actively moving their arms in a goal-oriented manner. Hence, first, the amount and nature of sensory information processed in two scenarios might weigh ownership differently and, second, two methods implicitly bias the attention to either the virtual hand (Grechuta et al., 2017) or the motor task (such as in the present experiment). We believe that future work is necessary to shed light on the effects of ownership on performance in a comparable setup.

## Conclusion

In conclusion, our results support the notion that the plasticity of body ownership depends on an active interplay between the experience-driven top-down predictions and bottom-up prediction errors driven by external and action-independent cues which pertain to the environment. Hence, these findings extend current accounts by demonstrating that the sensory evidence necessary for constructing ownership goes beyond the body and the PPS (Makin et al., 2008). In line with the motor control and perception studies, our data support a functional coupling between the predictive (generative) models of the body and environment (Miall and Wolpert, 1996). Moreover, our results are consistent with previous findings which demonstrate that body ownership, indeed, affects the perception of certain aspects of the environment (i.e., size of objects) (Van Dam and Stephens, 2018), which suggests a bidirectional link between the internal models of the environment and the body. Future work should provide a systematic study of the weighting of specific exafferent and reafferent unimodal and multisensory information in modulating the experience of body ownership under different tasks, performed by healthy participants and patients, as well as their neural underpinnings. Such analysis would shed light on which of the task components and the associated self or externally generated, proximal or distal, signals determine the plasticity of ownership in healthy and lesioned brains. At this point, however, we expect that the current findings will allow for the advancement of our understanding of the principles underlying the emergence and experience of body ownership, which we propose can be understood in a framework of the Bayesian framework of inference of all the signals within and outside of the PPS. We believe that the reported results can also contribute to the development of robust computer-based paradigms for educational purposes as well as for the treatment of neurological disorders of cognitive, perceptual, and motor functions in which embodiment plays a critical role.

## Data Availability Statement

The raw data supporting the conclusions of this article will be made available by the authors, without undue reservation.

## Ethics Statement

The studies involving human participants were reviewed and approved by the University Pompeu Fabra. The patients/participants provided their written informed consent to participate in this study.

## Author Contributions

KG, JD, and BB designed the protocol. JD conceived and conducted the experiment. KG and JD analyzed the results. KG, JD, BB, and PV wrote the manuscript. PV initiated and supervised the research. All authors reviewed and approved the manuscript.

## Conflict of Interest

PV is the founder and interim CEO of Eodyne SL, which aims at bringing scientifically validated neurorehabilitation technology to society. The remaining authors declare that the research was conducted in the absence of any commercial or financial relationships that could be construed as a potential conflict of interest.

## Publisher’s Note

All claims expressed in this article are solely those of the authors and do not necessarily represent those of their affiliated organizations, or those of the publisher, the editors and the reviewers. Any product that may be evaluated in this article, or claim that may be made by its manufacturer, is not guaranteed or endorsed by the publisher.

## Footnotes

1
www.vive.com

2
http://www.python.org
