# Supplementary material for: Challenging the Boundaries of the Physical Self: Distal Cues Impact Body Ownership
Source: Front Hum Neurosci. 2021 Oct 14;15:704414. doi: 10.3389/fnhum.2021.704414 (PMC8551865; doi:10.3389/fnhum.2021.704414)
Supplement: Supplementary file 1 [file Data_Sheet_1.docx]

**Virtual Hand**

The images of the virtual arm and hand are presented below, respectively.


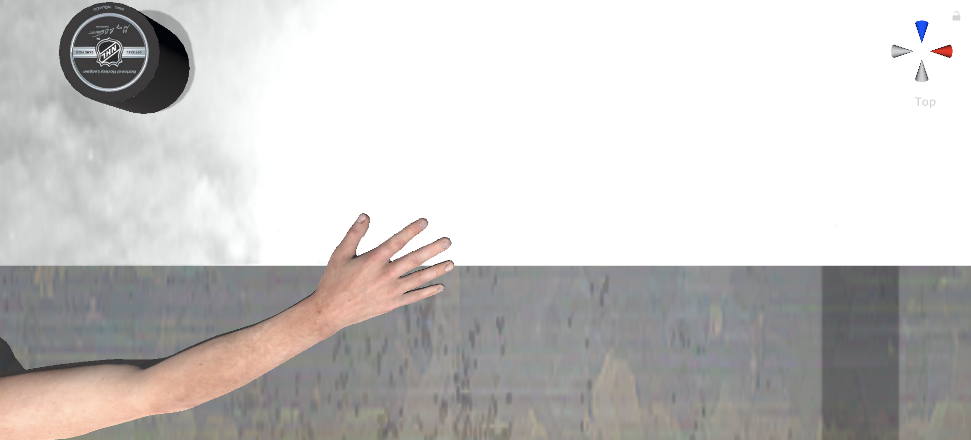

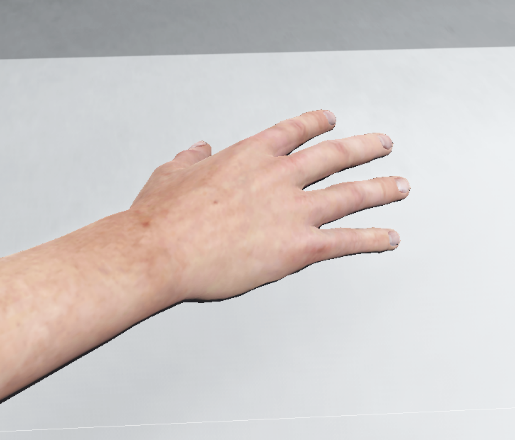


**Control measures**

Presence questionnaire We used this tool to evaluate whether subjects felt immersed in the proposed virtual environment. All questionnaire items are listed in Table 1. The answers for each statement are to be provided on a 7-point Likert Scale ranging from ‘-3’: being in strong disagreement to ‘3’: being in strong agreement.

Table 1 All items from presence questionnaire.

**
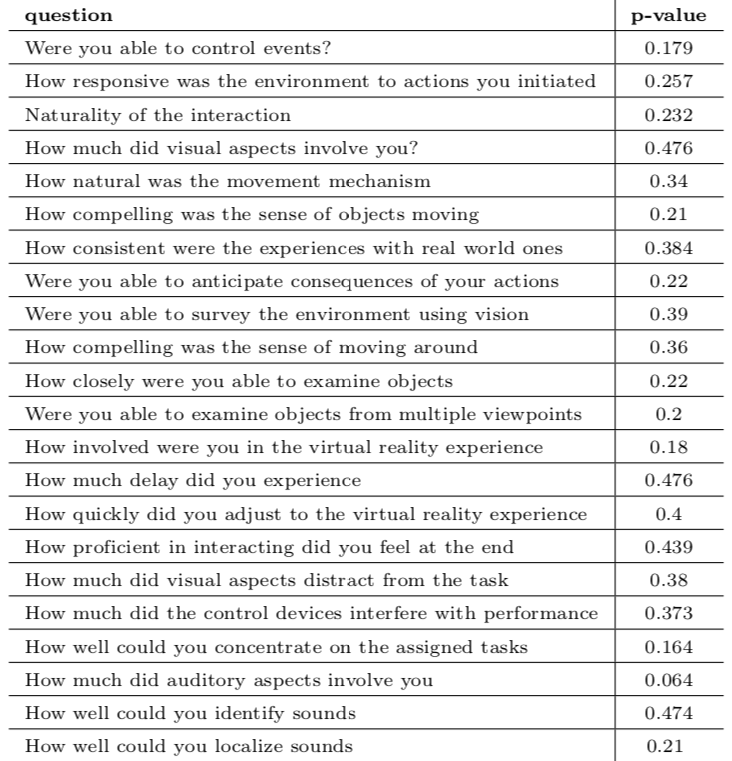
**

*Agency questionnaire* We used this tool to evaluate whether subjects felt agency over the virtual avatar. All questionnaire items are listed in *Table 2*. *The answers* for each statement *are to be provided on a* 7-point Likert Scale ranging from ‘-3’: being in strong disagreement to ‘3’: being in strong agreement*.*

Table 2 All items from presence questionnaire.


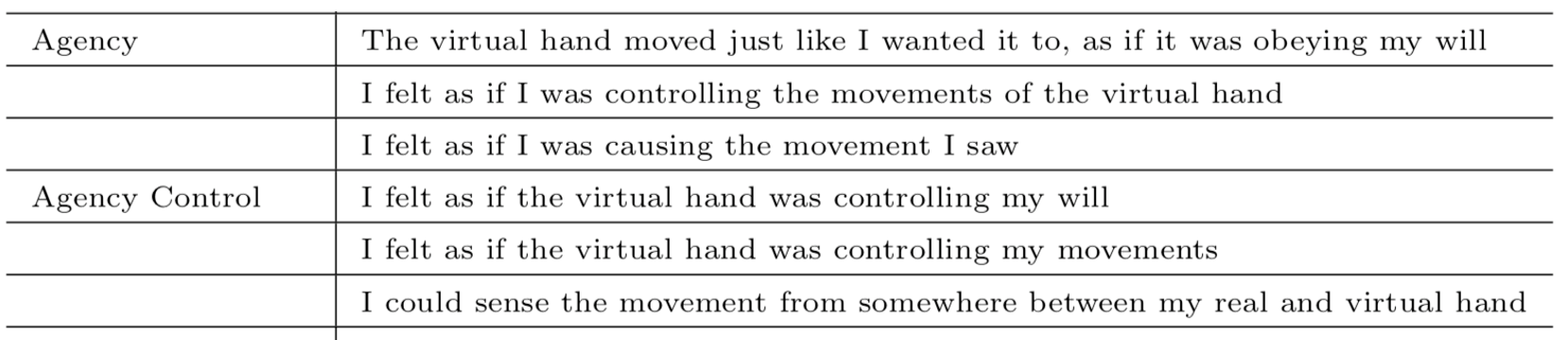


**Original analysis of hand withdrawal**

Initially, to quantify the execution of instinctive defensive movements in response to the unexpected Threatening Event (i.e., the virtual knife stabbing the virtual hand), we computed the velocity of displacement of the right virtual hand as a difference in the cumulative sum of the X (forward and backward) and Y (up and down) positions at every time step. The results were compared between the two conditions.

We observed that in the congruent condition participants exhibited faster velocity of the right virtual hand displacement post Threatening Event (hand withdrawal, Figure 1). In particular, the statistical analysis revealed that the difference between the incongruent and the congruent condition in the cumulative sum of the X and Y position over time (i.e., velocity) reached statistical significance at second 4 post Threatening Event (Figure 1, Right panel, p = 0.04).


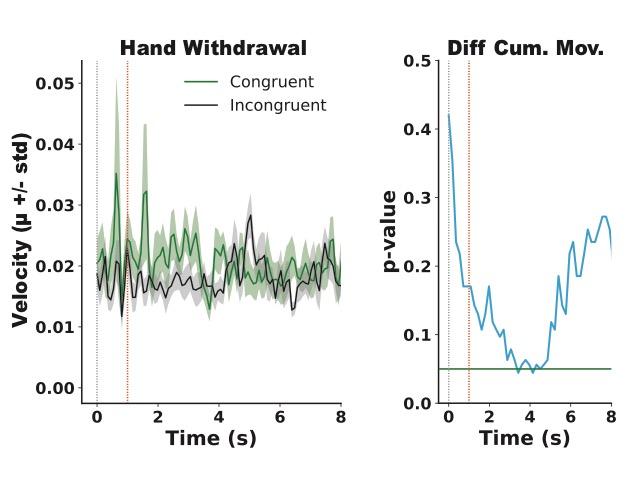


*Figure 1 Hand withdrawal results. Left. Changes in Movement Velocity Post-Threatening Event. The graph represents the evolution of changes in X and Y positions of the right virtual hand following the Threatening Event for each group over time. The data on the X-axis was recorded at 33Hz, and it is presented in an overlapping window of 66ms. Right. Changes in Velocity Between the groups. The blue line represents p − values obtained from Mann-Whitney U tests performed on the cumulative sum of the movement velocity. The values corresponding to each time step on the X-axis are shown in overlapping windows of 150-ms. In B, the green horizontal line indicates 0.05 significance threshold. In both graphs, the dashed grey line indicates the time when the knife appeared (time= 0) while the dashed red line shows the time when the knife stabbed the hand (time= 1).*
